# A Time-Series Study for Effects of Ozone on Respiratory Mortality and Cardiovascular Mortality in Nanchang, Jiangxi Province, China

**DOI:** 10.3389/fpubh.2022.864537

**Published:** 2022-04-26

**Authors:** Hao Wu, Keke Lu, Junjie Fu

**Affiliations:** ^1^Jiangxi Provincial Center for Disease Control and Prevention, Jiangxi, China; ^2^School of Public Health, Nanchang University, Jiangxi, China

**Keywords:** ozone pollution, cardiovascular mortality, respiratory mortality, time-series analysis, stratified analysis

## Abstract

**Objective:**

Most evidence comes from studies show that ambient ozone(O_3_) pollution has become a big issue in China. Few studies have investigated the impact of ozone spatiotemporal patterns on respiratory mortality and cardiovascular mortality in Nanchang city. Thus, this study aimed to explore the health effect of ozone exposure on respiratory mortality and cardiovascular mortality in Nanchang, Jiangxi Province.

**Methods:**

Using the daily mortality data, atmospheric routine monitoring data and meteorological data in Nanchang from 2014 to 2020, we performed a generalized additive model (GAM) based on the poisson distribution in which time-series analysis to calculate the risk correlation between respiratory mortality and cardiovascular mortality and ozone exposure level (8h average ozone concentration, O_3_-8h). Besides, analyses were also stratified by season, age and sex.

**Results:**

In the single-pollutant model, for every 10 μg/m^3^ increase in ozone, respiratory mortality increased 1.04% with 95% confidence interval (CI) between 0.04 and 1.68%, and cardiovascular mortality increased 1.26% (95%CI: 0.68 ~ 1.83%). In the multi-day moving average lag model, the mortality of respiratory diseases and cardiovascular diseases reached a relative risk peak on the cumulative lag5 (1.77%,95%CI: 0.99 ~ 2.57%) and the cumulative lag3 (1.68%,95%CI: 0.93 ~ 2.45%), respectively. The differences were statistically significant (*P* < 0.05). Results of the stratified analyses showed the effect value of respiratory mortality in people aged ≥65 years was higher than aged <65 years, whereas the greatest effect of cardiovascular mortality in people aged <65 years than aged ≥65 years. Ozone had a more profound impact on females than males in respiratory diseases and cardiovascular diseases. In winter and spring, ozone had a obvious impact on respiratory mortality, and effects of ozone pollution on cardiovascular mortality were stronger in summer and winter. There was a statistically significant difference of respiratory mortality in winter and spring and of cardiovascular mortality in summer and winter (*P* < 0.05).

**Conclusions:**

In the long run, the more extreme the pollution of ozone exposure, the higher the health risk of residents' respiratory mortality and cardiovascular mortality. Therefore, the government should play an important role in the prevention and control ways of decreasing and eliminating the ozone pollution to protect the resident's health. The findings provide valuable data for further scientific research and improving environmental policies in Nanchang city.

## Introduction

With the rapid development of modern industrialization, the air quality around us is increasingly affected (1). The World Health Organization (WHO) estimated that air pollution causes the deaths of 4.2 million people each year, which accounts for 6% of total deaths worldwide (2). Based on the analysis of atmospheric environment in 1,600 cities and regions around the world, it was found that only 12% of the study areas met the safety standards stipulated by WHO (3), indicating that the status and development of global air pollution cannot be ignored. Since the Chinese government implemented China's Action Plan of Prevention and Control of Air Pollution in 2013, atmospheric particulate matter has dropped significantly (4). Nevertheless, along with the rapid development of urban basic infrastructure and growth in motor vehicle number, ozone concentration has dramatically increased recently (5). Ozone is a secondary pollutant, its formation is due to the interaction between the hydrocarbons and oxides of nitrogen released from car exhaust and sunlight (UV), leading to photochemical smog. It can affect air quality at local, regional and even global scales, beside, it has an important impact on animal health, plant growth, climate change and global ecological balance. At the same time, the adverse effects on human life and ecological environment are gradually increasing (6). The more serious the air pollution is, the more the residents' medical expenses will increase (7).

According to the 2020 China Ecological Environment Bulletin, ozone has been become an important pollutant besides particulate matter (PM_2.5_, PM_10_) at the national level (8). Compared with Japan, South Korea, Europe and the United States, the magnitude and frequency of high-ozone events in China are much more significant (9). China experiences high ozone concentrations with highest annual 8-h maximum concentration in eastern China of 78 μg/m^3^ and was followed by southern (73μg/m^3^), north-western (69μg/m^3^), northern (68μg/m^3^), central (67μg/m^3^), north eastern (65μg/m^3^) and south-western China (59μg/m^3^) (10). The majority of the Chinese population lives in the eastern part of China, especially in the three most developed regions, Jing-Jin-Ji (Beijing-Tianjin-Hebei), the Yangtze River Delta (including Shanghai-Jiangsu -Zhejiang-Anhui), and the Pearl River Delta (including Guangzhou, Shenzhen, and Hong Kong). These regions consistently have the highest emissions of anthropogenic precursors, which have led to severe region-wide air pollution (11). Studies showed that according to monitoring results from 74 Chinese cities, the mean daily 8-h maximum concentrations of ozone increased from approximately 69.5 ppbv in 2013 to 75.0 ppbv in 2015, while the percentage of non-compliant cities increased from 23 to 38% (5, 10). Jiangxi Province lies on the southern bank of the Yangtze River's lower and middle sections, it has a sub-tropical climate, warm and humid. Kan (12) using the data of environmental monitoring stations data in Jiangxi Province from January 2017 to June 2020, showed that from 2017 to 2019, the time and mass concentration of ozone exceeding the limit showed an increasing trend year by year. April to June and August to October were the periods of high incidence of ozone pollution. Nanchang City is the Central City of Jiangxi Province, and it is one of the first cities to implement new ambient air quality standards. The daily 8-hour maximum concentration of ozone in Nanchang from 2013 to 2015 was analyzed, showing that the seasonal variation of ozone was high in spring and summer and low in autumn and winter (13).

Many epidemiological studies had been conducted to assess the characteristics of ozone pollution exposure in China, related research showed that the concentration of ozone in Yangtze River Delta, Beijing-Tianjin-Hebei, Pearl River Delta and Chengdu-Chongqing cities was increasing year by year (14–17). There is a growing interest in studying the potential effect of ozone levels, and its subsequent effects on public health. Epidemiological and toxicological studies had shown that ozone exposure is not only associated with adverse health outcomes (18–22), but also leads to significant economic burdens (23). A series of epidemiological studies reported that short-term ozone exposure is strongly associated with the death risk of cardiovascular diseases (24, 25) and respiratory diseases (26). China recorded 93,351 (95%CI: 11,001–169,898) ozone related premature mortality in 2015 with 42,673 (95%CI: 11,001-69,586) respiratory mortality and 50,678 (95%CI: 0–100,312) cardiovascular mortality. Northern and eastern China recorded high ozone related mortality with 18,230 (95%CI: 4,700–29,727), 12,261 (95%CI: 3,161–19,993) respiratory, 21,662 (95%CI: 0–42 877) and 14,528 (95%CI:0–28 757) cardiovascular deaths respectively (11). Kamal and Anil (27) showed that the proportion of all-cause, cardiovascular and respiratory premature deaths attributed to short-term environmental ozone exposure in China in 2019 increased by 19.6, 19.8, and 21.2% in comparison with those in 2015. Ozone is one of the most powerful oxidizing molecule to which living beings can be exposed. Accordingly, ozone inhalation may cause oxidative damages and inflammation, which could expand from the respiratory system to the periphery and to the brain, for which the nose and olfactory pathway is another portal of entry (28). Animal and human exposure studies had been proved that ozone has a stimulating effect on human mucosa through the eyes, nose, and mouth into the lungs, which will also cause damage to the lung tissue. Therefore, excessive ozone concentration will increase the probability of human suffering from respiratory diseases, and also aggravate the condition of patients with respiratory diseases such as asthma and chronic lung diseases (29). Some scholars pointed out that Short-term ozone exposure at levels was associated with platelet activation and blood pressure increases, suggesting a possible mechanism by which ozone may affect cardiovascular health (30). The proposed mechanisms include systemic inflammation and oxidative stress, autonomic nervous system imbalance, and abnormal epigenetic changes (31).

In areas with good air quality, ozone has gradually become the main pollution factor affecting the air quality compliance rate, which is closely related to climate change, and its composition is complex and difficult to control. In the preparation of the “14th Five-Year Plan” ecological and environmental protection plan, it is necessary to pay attention to the coordinated treatment of PM_2.5_ and ozone in view of the prominent problems of ozone pollution (32). Among China's 2030 climate target, as the largest developing country in the world, China has overcome its own economic and social difficulties, implemented a series of strategies, measures and actions to cope with climate change, participated in global climate governance, and achieved positive results in addressing climate change. China has always attached great importance to non-carbon dioxide greenhouse gas emissions and China has accepted the Kigali Amendment to the Montreal Protocol on Substances that Deplete the Ozone Layer, which has entered a new stage in protecting the ozone layer and responding to climate change (33). Nitrogen oxides (NOx) and volatile organic compounds (VOCs) are important precursors to ozone formation. In order to control ozone pollution during the “14th Five-Year Plan” period, Jiangxi will promote the energy structure, adjustment and optimization of industrial structure and traffic structure to reduce NOx and VOCs emissions from the source (34). Nanchang is an important central city in the middle and lower reaches of the Yangtze River in China, due to the city is a lack of research on the effects of ozone pollution, and the association of human health with ambient ozone have not been fully understood. Therefore, the main objective of this study is to evaluate the association between ozone exposure and respiratory mortality and cardiovascular mortality, at the same time, we aimed to evaluate individual characteristics (sex and age) and season as potential effect modifiers of the ozone-mortality association, which providing a basis for further research and formulation of local environmental prevention and control policies in Nanchang City.

## Materials and Methods

### Study Area

Nanchang is located in the north-central part of Jiangxi Province, at the end of the Ganjiang River and the Fuhe River. It is the capital of Jiangxi Province and is considered to be a provincial political, economic, cultural, scientific, technological and information center. Importantly, it is the core city of the Poyang Lake Ecological Economic Zone, which promotes the economic development and urbanization of the province (35). The study area is a typical subtropical monsoon climate with mild climate, sufficient sunshine, abundant rainfall and four distinct seasons with a long frost-free period, spring and autumn are short, while winter and summer are long. In recent years, with the optimization of economic structure and industrial overall layout of Nanchang, people's living standards have improved significantly, but also brought a series of air pollution problems.

### Data Collection

The daily mortality data of the respiratory diseases and cardiovascular diseases in Nanchang from 2014 to 2020 were selected, included sex, age, death time and underlying cause of death code (ICD-10), which were cardiovascular diseases death (I05–I52) and respiratory diseases death (J00–J99), respectively. The data were derived from the death cause registration system of Chinese Center for Disease Control and Prevention. Atmospheric pollutant data and meteorological data of Nanchang City are from the Nanchang Environmental Monitoring Center and Meteorological Bureau of Nanchang Municipality during the same period, respectively. Atmospheric pollutants included O_3_-8h(μg/m^3^), SO_2_(μg/m^3^), NO_2_(μg/m^3^), PM_2.5_(μg/m^3^), PM_10_(μg/m^3^), CO(mg/m^3^), which is the arithmetic mean of the data from eight nationally controlled monitoring sites in Nanchang city; meteorological data included daily average temperature (°C), daily average relative humidity (%), daily average air pressure (kPa) and daily average wind speed (m/s). The World Health Organization proposed that daily 8-h maximum ozone concentration as an appropriate indicator to study the relationship between environmental ozone exposure and health in 2020 (36). It is usually divided into four seasons according to the position of the earth around the solar orbit. In China, March to May is generally counted as spring (MMA), June to August as summer (JJA), September to November as autumn (SON), and December to February as winter (DJF).

### Time-Series Analysis

Generalized additive model based on Quasi-Poisson regression was used to estimate the correlation between O_3_-8h exposure and respiratory mortality and cardiovascular mortality in Nanchang city. The model was specified as follows:


(1) $$\begin{array}{l} \text{Log}\left[\text{E}\left({\text{y}}_{\text{i}}\right)\right]=\alpha +{\text{β}}_{\text{i}}{\text{X}}_{\text{i}}+\text{ns}\left(\text{time},\text{df}\right)+\text{ns}\left({\text{Z}}_{\text{i}},\text{df}\right)\\ \;+\text{DOW}+\text{PH} \end{array}$$

In this equation, i refers to the day of the observation; E (y_i_) is the expected number of non-accidental mortality of residents on day i; α is the intercept; x_i_ refers to the concentration level of O_3_-8h (μg/m^3^) on day i; βi represents the regression coefficient of the corresponding air pollutants; ns is the natural smoothing spline function, and df represents the degree of freedom. Previous studies have usually set the degrees of freedom of time to 5 to 7 and meteorological factors to 6 (22, 37); DOW as the variables of weeks; PH is the holiday effect; Zi as the meteorological factor of day i, including daily average temperature and daily average relative humidity; time is the date variable, the appropriate degree of freedom is selected by using the minimum sum of the absolute values of the partial autocorrelation function (PACF) of the basic model residual to effectively control the long-term and seasonal fluctuation trend of the pollution-death series data. The excess risk of non-accidental death (ER) caused by 10 μg/m^3^ increase of O_3_-8h concentration was calculated by formula (2).


(2) $$\begin{array}{l} \text{ER}=\left[\text{exp}\left({\text{β}}_{\text{i}}\times 10\right)-1\right]\times 100\text{\%} \end{array}$$

The O_3_-8h pollutant model was introduced to analyze the concentration of O_3_-8h on the same day (Lag0) and the number of non-accidental deaths of residents. Meanwhile, the lag effect and cumulative lag effect were analyzed. According to the significance of the model analysis results, Lag0-Lag7 was selected for analysis (38, 39). Lag0 represents the average concentration of O_3_-8h on the day and Lag1 represents the average concentration of O_3_-8h on the lag one day, and so on. Previous studies have shown that the cumulative effect of multi-day lag is greater than that of single-day lag of air pollutants (40, 41). Therefore, we further used the moving average of air pollutant concentrations from 2nd day to 8th day (lag01 to lag07) in the analysis, where Lag01 represents the moving average concentration of O_3_-8h on the current day and the previous day, Lag02 represents the moving average concentration of O_3_-8h on the current day and the previous 2 days, and so on.

### Statistical Analysis

The data did not conform to the normal distribution, so the median (M), quartile spacing (P_25_, P_75_), minimum and maximum values were used to describe the air pollutant level, meteorological factors and respiratory mortality and cardiovascular mortality in the study period. In addition, we conducted stratified analyses by age, sex and season. Spearman rank correlations were performed to evaluate the relationship between O_3_-8h and other atmospheric pollutants and meteorological conditions. Temporal changes of air pollutant were summarized by Origin 8.0 software; SPSS 23.0 was used to describe the air pollutants, meteorological factors and the number of daily mortality in respiratory and cardiovascular disease. Non-normal distributions are performed using independent sample nonparametric tests (Kruskal-WallisTest). The time-series statistical analysis was conducted using R software, version 4.1.2. The statistical significance of all analyses was set as *P* < 0.05.

## Results

### Overview of Air Pollution in Study Area

**Figure 1:**
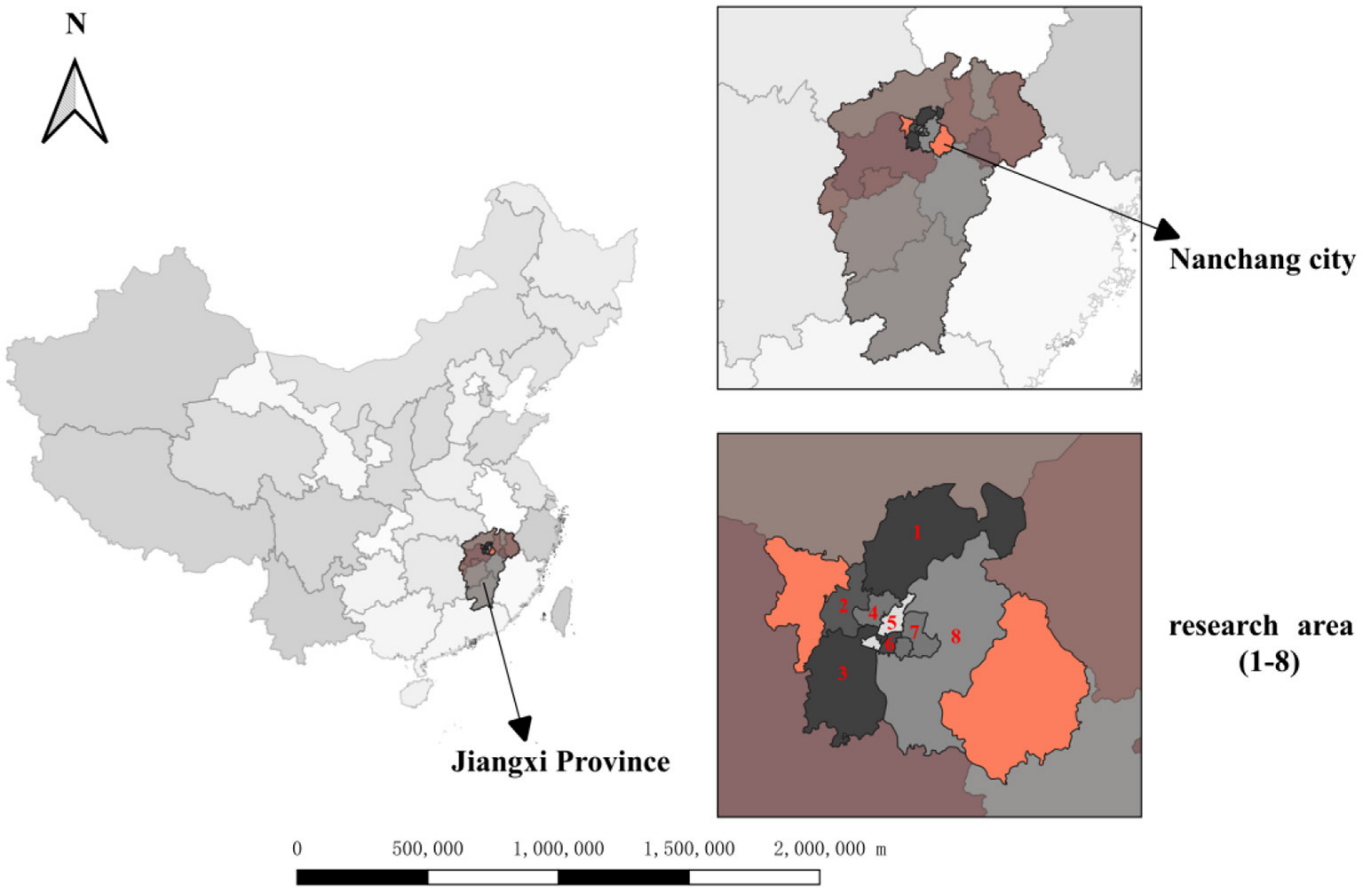
Ozone monitoring sites in Nanchang city, Jiangxi province, 2014–2020.

**Figure 2:**
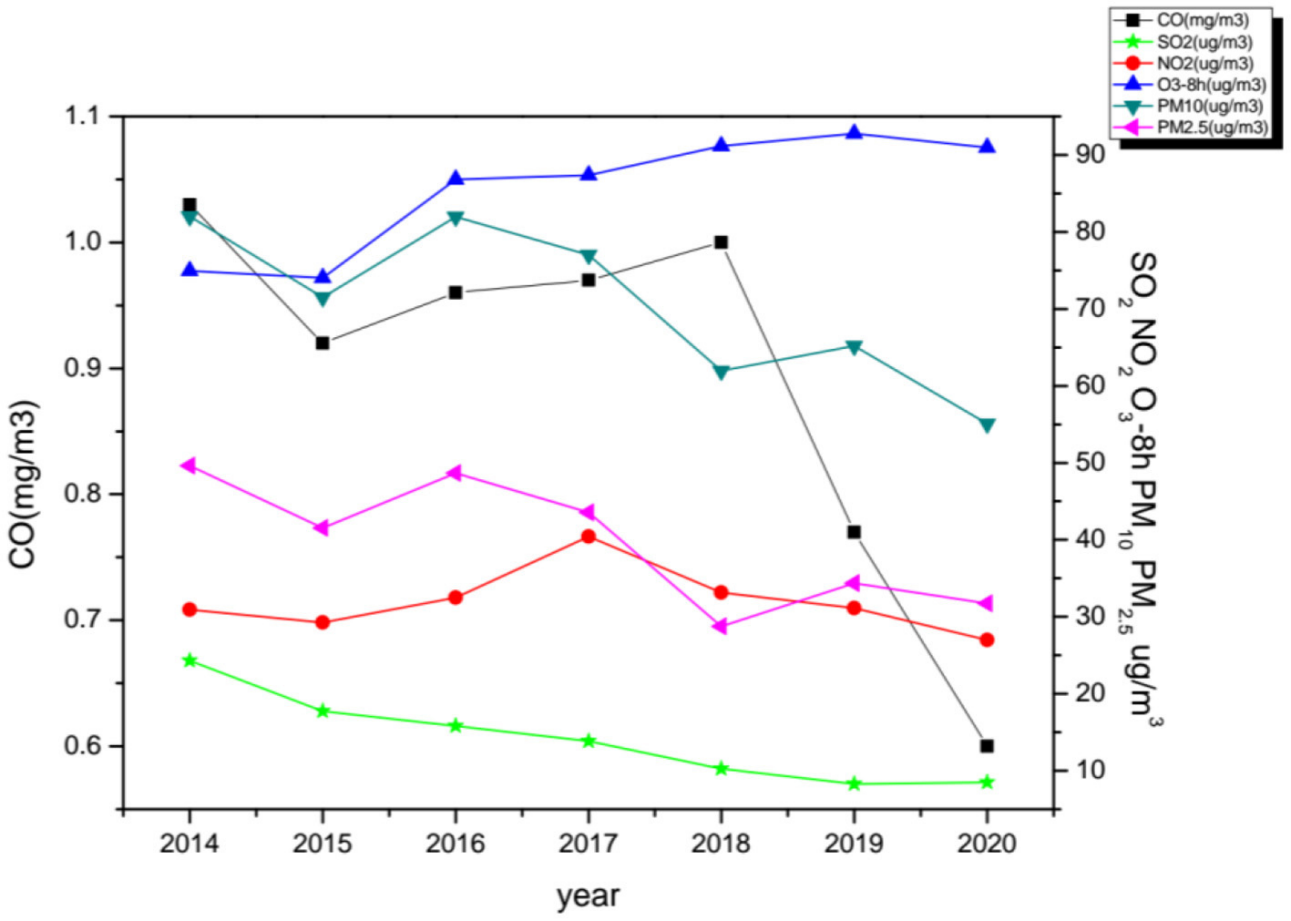
The annual change trend of air pollutants in Nanchang city, 2014–2020.

From 2014 to 2020, we monitored eight ozone monitoring sites and obtained ozone concentration and meteorological data in Nanchang City (Figure 1). During the study period, the average annual concentrations of PM_2.5_, PM_10_, and SO_2_ all showed a steady downward trend, from 49.63 μg/m^3^, 82.02 μg/m^3^, 24.32 μg/m^3^ in 2014 to 31.75 μg/m^3^, 55.07 μg/m^3^, 8.49 μg/m^3^ in 2020 in Nanchang City. The average annual concentration of NO_2_ increased firstly, reached the peak in 2017 (40.44 μg/m^3^) and then began to decline, and fell to 26.98 μg/m^3^ in 2020. The annual concentration of CO increased firstly, then decreased rapidly to 0.6 mg/m^3^ in 2020. Among the six air pollutants, the average annual concentration of O_3_-8h is increasing year by year, reaching 90.98 μg/m^3^ in 2020 (Figure 2).

### The Basic Situation of Air Pollution, Meteorological Factors and Daily Mortality

The number of mortality from respiratory diseases was 28,938, with 19,015 males and 9,920 females, accounting for 65.7 and 34.3%, respectively. The number of respiratory mortality in people aged ≥65 years was 23,943 and aged <65 years was 4,993, accounting for 82.7 and 17.3%, respectively. Besides, There were 29,481 deaths of cardiovascular diseases, 15,457 deaths of males and 14,002 deaths of females, accounting for 52.4 and 47.6%, respectively. The number of cardiovascular mortality in people aged ≥65 years was 25,757 and aged <65 years was 3,703, accounting for 87.4 and 12.6%, respectively. The median of daily respiratory mortality was 11 (8–14) and cardiovascular mortality was 11 (8–15). The daily median concentrations of SO_2_, NO_2_, O_3_-8h, CO, PM_2.5_ and PM_10_ were 11.50 (7.27–18.67)μg/m^3^, 28.15 (21.20–39.08)μg/m^3^, 85.00 (55.00–115.23)μg/m^3^, 0.89 (0.70–1.08)mg/m^3^, 33.00 (21.00–50.67)μg/m^3^, 61.14 (39.66–91.77)μg/m^3^, respectively. During the research period in Nanchang city, the median of daily average air pressure, daily average temperature, daily average relative humidity and daily average wind speed were 1,009.80 (1,002.20–1,016.85) hpa, 20.20 (11.40–26.30)°C, 75.00 (64.80–85.00)% and 1.70 (1.30–2.40)m/s, respectively. As important meteorological factors affecting atmospheric pollutants, daily average temperature and daily average relative humidity are significantly different in four seasons. The maximum of daily average relative humidity in spring is 77.65 (66.00–86.45)%, whereas the minimum of daily average relative humidity is 73.00 (62.55–83.00)% in autumn. Besides, the median of average temperature in summer is the highest, that is 29.00 (26.50–31.18)°C, and the median of average temperature in winter is the lowest, the value is 8.10 (5.90–10.30)°C. The distribution characteristics of atmospheric pollutants, meteorological indicators and daily mortality of respiratory and cardiovascular disease in Nanchang was shown in Table 1.

**Table 1:** Distribution characteristics of air pollutants, meteorological indicators and non-accidental deaths in Nanchang City, 2014–2020.

**Variables**	**Min**	**P_**25**_**	**M**	**P_**75**_**	**Max**
**Air pollutants**					
SO_2_ (μg/m^3^)	2.00	7.27	11.50	18.67	78.50
NO_2_ (μg/m^3^)	6.10	21.20	28.15	39.08	123.00
CO (mg/m^3^)	0.20	0.70	0.89	1.08	2.70
O_3_-8h (μg/m^3^)	5.70	55.00	85.00	115.23	217.40
PM_10_ (μg/m^3^)	5.20	39.66	61.14	91.77	362.20
PM_2.5_ (μg/m^3^)	4.00	21.00	33.00	50.67	304.00
**Meteorological indicators**					
Daily mean air pressure (hpa)	989.00	1,002.20	1,009.80	1,016.85	1,038.20
Daily mean wind speed (m/s)	0.40	1.30	1.70	2.40	10.00
Daily mean relative humidity (%)	31.00	64.80	75.00	85.00	100.00
Spring (MAM)	34.00	66.00	77.65	86.45	98.00
Summer (JJA)	44.00	67.00	76.00	85.00	99.00
Autumn (SON)	33.50	62.55	73.00	83.00	100.00
Winter (DJF)	31.00	60.00	74.00	85.00	97.00
P	*P* < 0.05				
**Daily mean temperature (°C)**	−1.30	11.40	20.20	26.30	34.90
Spring (MAM)	3.70	14.40	19.50	23.10	31.60
Summer (JJA)	19.50	26.50	29.00	31.18	34.90
Autumn (SON)	4.00	16.50	20.50	24.90	31.80
Winter (DJF)	−1.30	5.90	8.10	10.30	22.30
P	*P* < 0.05				
**Daily mortality counts of respiratory disease**					
All	1	8	11	14	38
Male	0	5	7	10	24
Female	0	2	3	5	21
≥65	0	6	9	12	32
<65	0	1	2	3	11
**Daily mortality counts of cardiovascular disease**					
All	1	8	11	15	42
Male	0	4	6	8	24
Female	0	3	5	7	21
≥65	0	7	9	13	40
<65	0	0	1	2	12

Moreover, the Spearman correlation analysis in Table 2 showed that O_3_-8h exposure is positively correlated with NO_2_ and CO (*P* < 0.05), but not related to PM_2.5_ (*P* > 0.05). It is negatively correlated with daily average air pressure, daily average relative humidity, and precipitation, whereas positively correlated with daily average temperature, but has nothing to do with daily average wind speed. It was remarkably observed that meteorological indicators are one of the significant factors affecting ozone pollution.

**Table 2:** Correlation analysis of air pollutants and meteorological indicators in Nanchang city.

**O_**3**_-8h (μg/m^**3**^)**	**Air pollutants**				**Meteorological conditions**			
	**NO_**2**_ (μg/m^**3**^)**	**CO (mg/m^**3**^)**	**PM_**10**_ (μg/m^**3**^)**	**PM_**2.5**_ (μg/m^**3**^)**	**Daily mean air pressure (hpa)**	**Daily mean temperature (°C)**	**Daily mean relative humidity (%)**	**Daily mean wind speed (m/s)**
r	−0.096	−0.14	0.282	0.03	−0.398	0.585	−0.573	0.053
P	<0.05	<0.05	<0.05	>0.05	<0.05	<0.05	<0.05	>0.05

### The Health Effect of O_3_ Exposure

**Figure 3:**
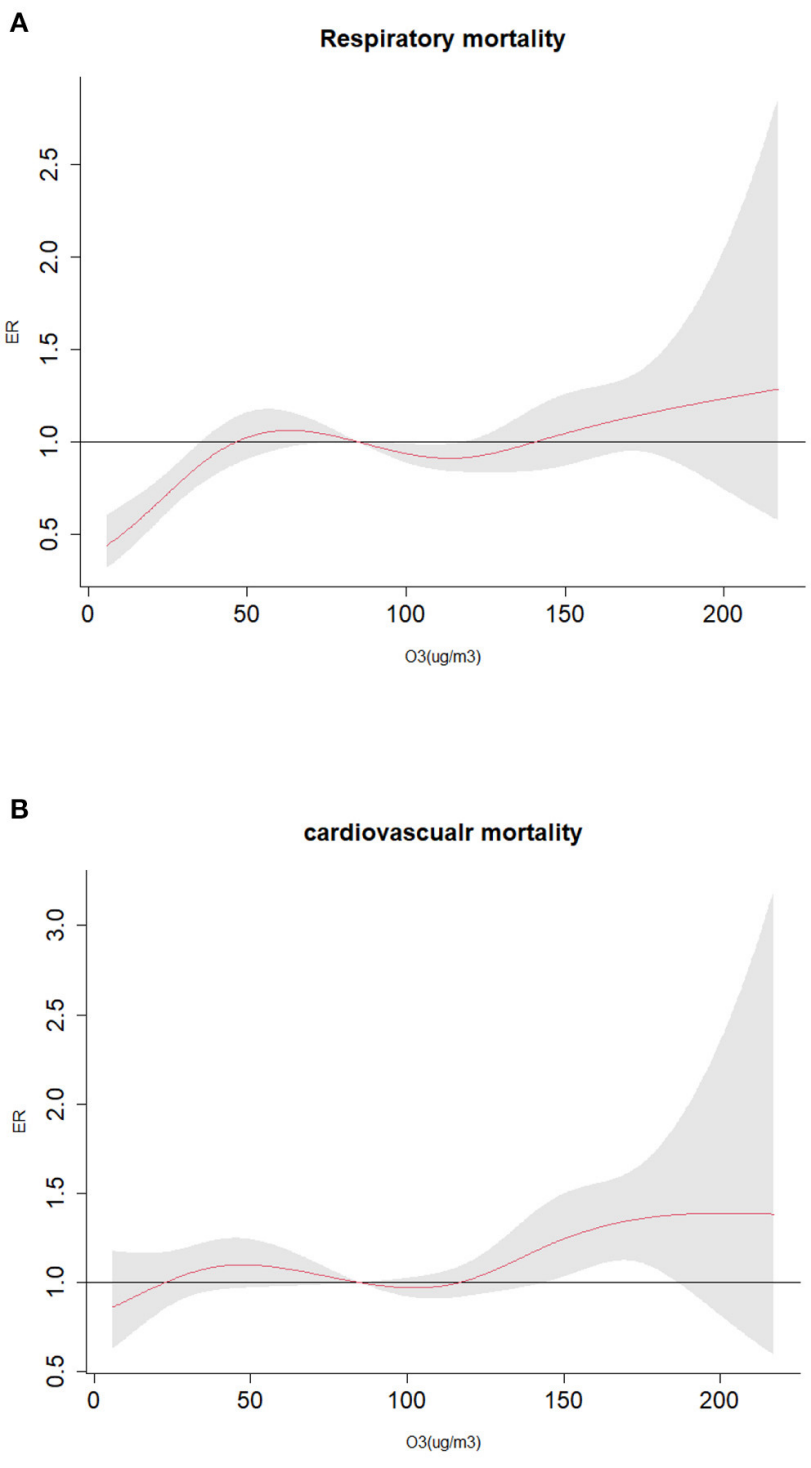
Exposure-response curve of O_3_-8h to major death causes of residents; **(A)** O_3_-8h lead to respiratory mortality; **(B)** O_3_-8h lead to cardiovascular mortality.

**Figure 4:**
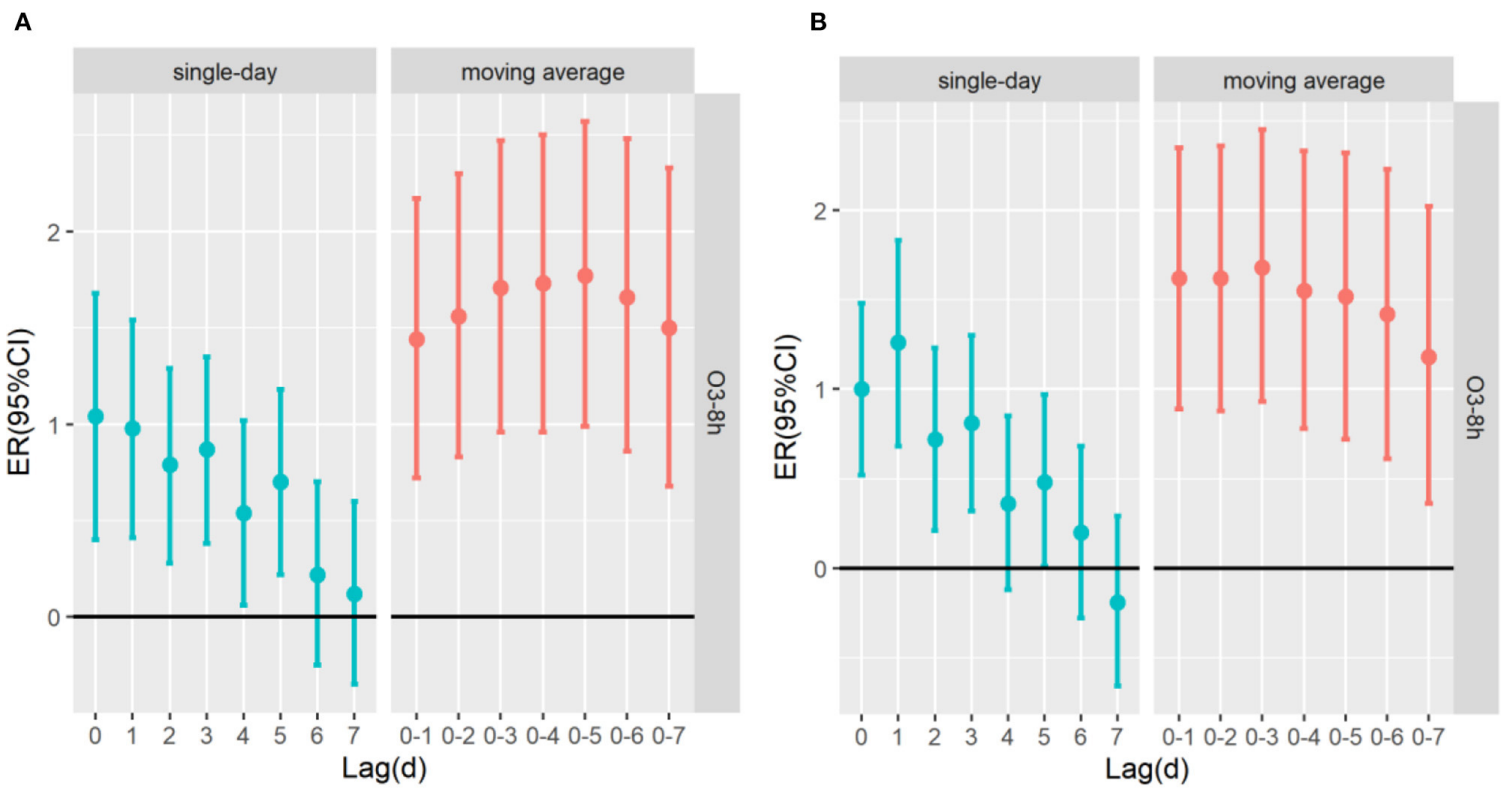
The ER (95%CI) of mortality associated with 10 μg/m^3^ increase of O_3_-8h concentration; **(A)** O_3_-8h lead to respiratory mortality; **(B)** O_3_-8h lead to cardiovascular mortality.

This study emphatically analyzed the effect of ozone exposure on respiratory mortality and cardiovascular mortality in different sex, age and season. By drawing the exposure-response relationship between ozone and the risk of death of residents in Figure 3, it can be seen that the increase of ozone concentration is positively correlated with the excessive risk of death on respiratory diseases and cardiovascular diseases. The influence curve of ozone on the death of residents is approximately linearly increasing, and there is no discernible threshold. At low concentrations, it also has a certain impact on the death of residents. Figure 4 indicates that the estimated lag structure of the effects of a 10 μg/m^3^ increase in O_3_-8h concentration on respiratory diseases and cardiovascular diseases for the whole group. After adjusting for time, meteorological factors, day of the week effects, holiday effects and other confounding factors, it is focused on that under different hysteresis models with the average concentration of O_3_-8h increase of 10μg/m^3^ leads to respiratory mortality and cardiovascular mortality (ER,95%CI). For the single lag model, the strongest effect of O_3_-8h on respiratory mortality and cardiovascular mortality on the current day (lag0) and 1th day (lag1), respectively, that is, for every 10μg/m^3^ increase in O_3_-8h, the risk of death of respiratory diseases and cardiovascular diseases increased by 1.04% (95%CI: 0.40–1.68%), 1.26%(95%CI: 0.68–1.8%), respectively. The multi-day moving average lag model showed that respiratory mortality and cardiovascular mortality were the highest on the 15 day (1.77%, 95%CI:0.99–2.57%) and the third day (1.68%,95%CI: 0.93–2.45%) of the cumulative lag, respectively. The differences were statistically significant (*P* < 0.05). To avoid multiple colinearities, only the two-pollutant model was used to detect the robustness of the model (42). Table 3 shows that in the two-pollutant models, the effect of O_3_-8h were still significantly after adding SO_2_, NO_2_, CO, PM_2.5_ and PM_10_ on respiratory mortality and cardiovascular mortality. Besides, the effect of respiratory mortality had no significant change, while the risk of death from cardiovascular diseases caused by O_3_-8h is reduced by 0.23, 0.23, 0.35, 0.38, and 0.27%, respectively.

**Table 3:** The excess risk (95%CI) of air pollutant associated with 10μg/m^3^ increase of O_3_-8h concentration in the double-pollutant models.

**Air pollutant models**	**ER (95%CI)**	
	**Respiratory mortality**	**Cardiovascular mortality**
**Single-pollutant model**		
O_3_-8h	1.04 (0.40,1.68)^*^	1.26 (0.68,1.83)^*^
**Two-pollutant models**		
O_3_-8h+SO_2_	1.05 (0.41,1.70)^*^	1.03 (0.38,1.68)^*^
O_3_-8h+NO_2_	1.03 (0.39,1.68)^*^	1.03 (0.38,1.68)^*^
O_3_-8h+PM_2.5_	0.99 (0.35,1.64)^*^	0.91 (0.26,1.56)^*^
O_3_-8h+PM_10_	0.96 (0.32,1.61)^*^	0.88 (0.23,1.54)^*^
O_3_-8h+CO	1.03 (0.39,1.68)^*^	0.99 (0.35,1.64)^*^

* *P < 0.05*.

**Figure 5:**
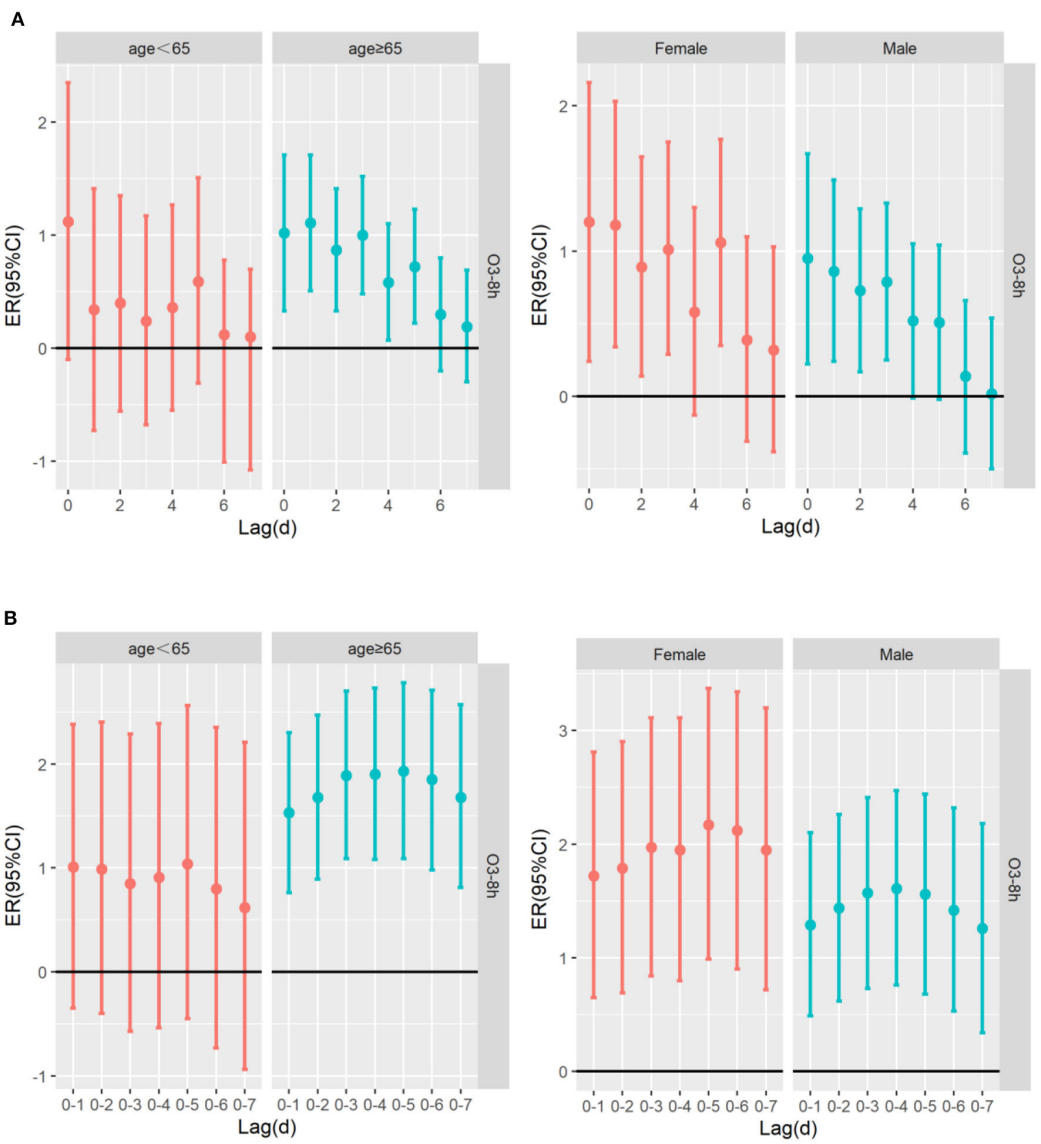
The ER (95%CI) of respiratory mortality in age and sex lag-response relationship associated with 10 μg/m^3^ increase of O_3_-8h concentration; **(A)** The risks of respiratory mortality associated with 10 μg/m^3^ increase of O_3_-8h; **(B)** The cumulative risks of respiratory mortality associated with 10 μg/m^3^ increase of O_3_-8h.

**Figure 6:**
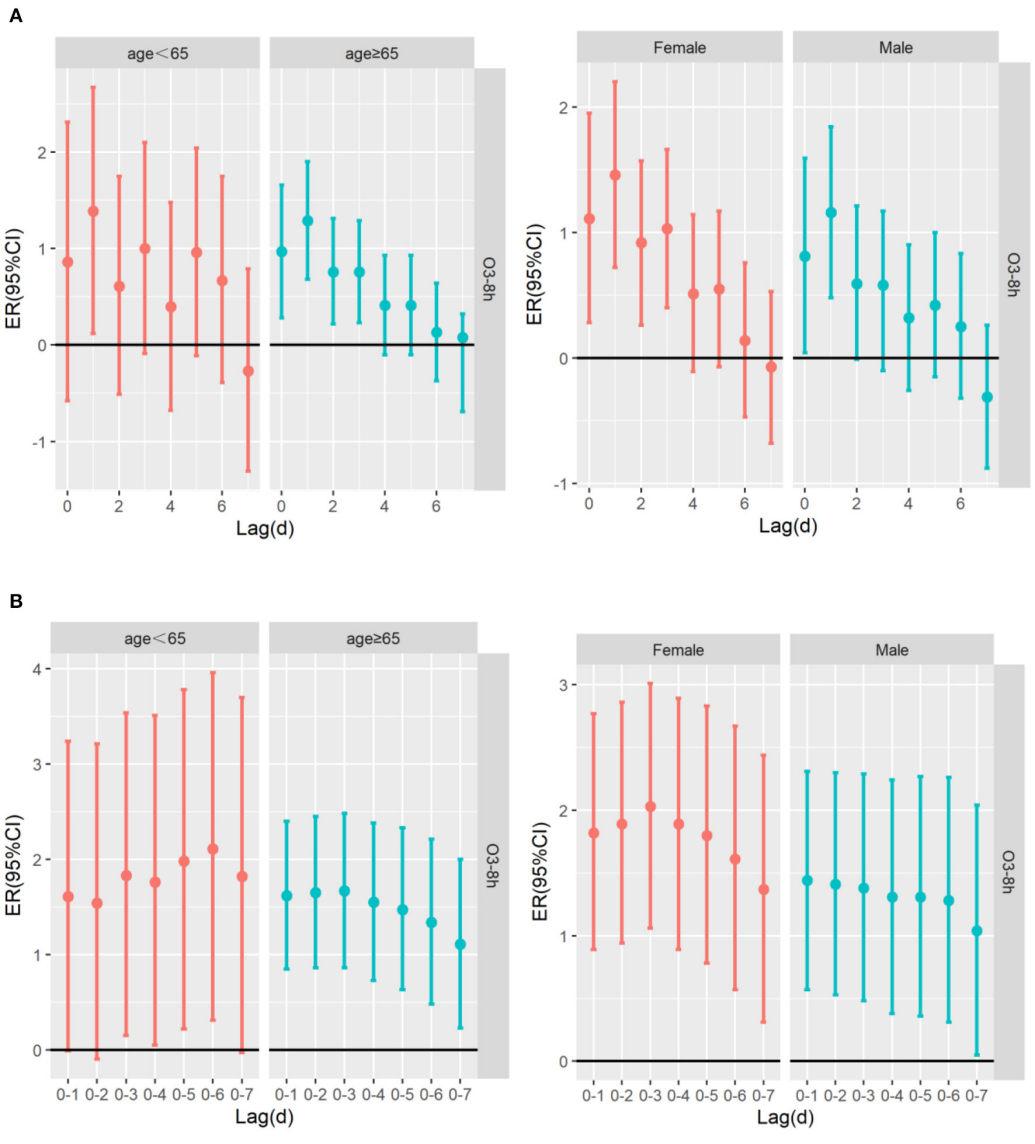
The ER (95%CI) of cardiovascular mortality in age and sex lag-response relationship associated with 10 μg/m^3^ increase of O_3_-8h concentration; **(A)** The risk of cardiovascular mortality associated with 10 μg/m^3^ increase of O_3_-8h; **(B)** The cumulative risks of cardiovascular mortality associated with 10 μg/m^3^ increase of O_3_-8h.

**Figure 7:**
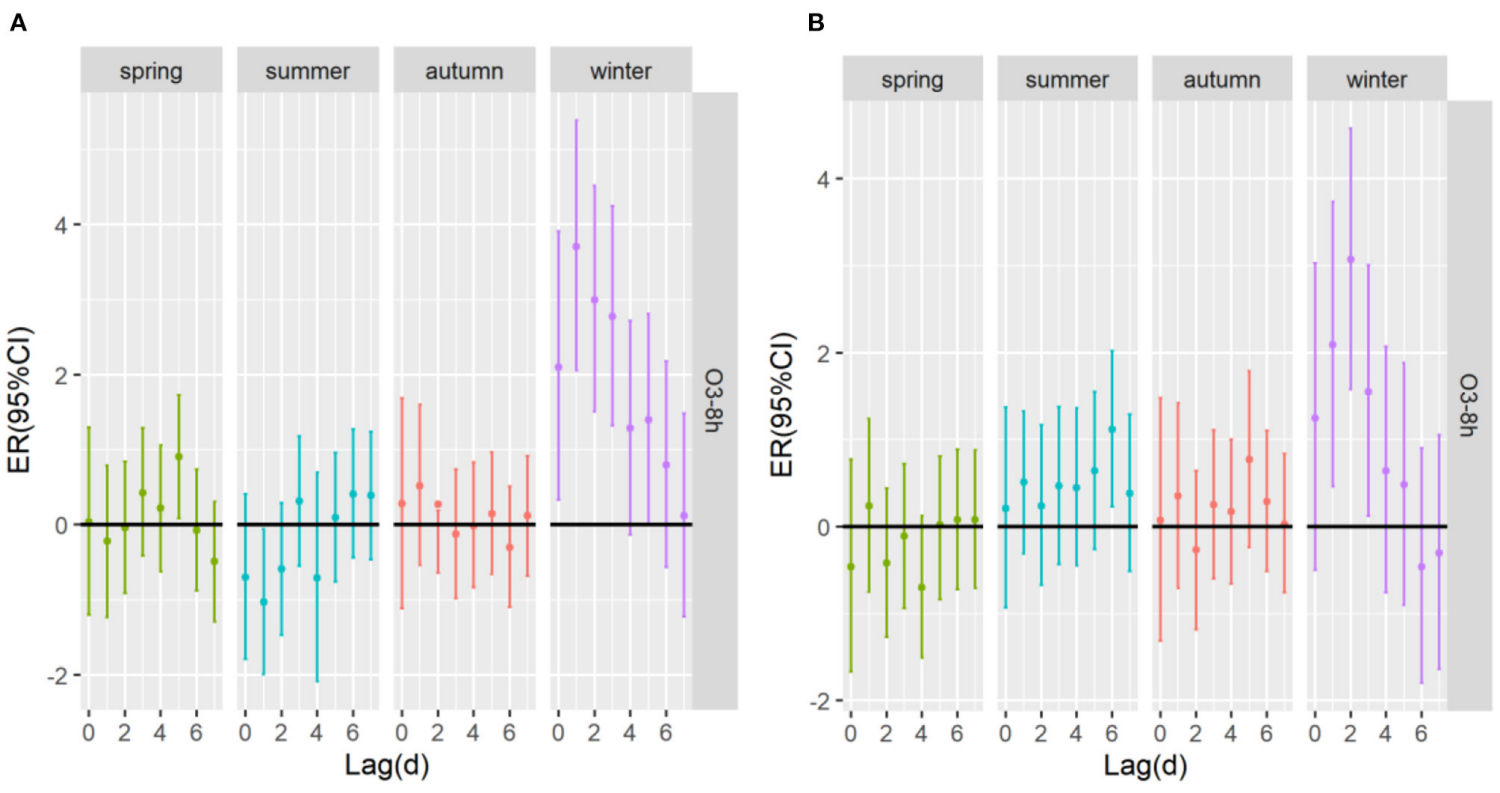
The ER (95%CI) of mortality in season lag-response relationship associated with 10 μg/m^3^ increase of O_3_-8h concentration; **(A)** O_3_-8h lead to respiratory mortality; **(B)** O_3_-8h lead to cardiovascular mortality.

**Figure 8:**
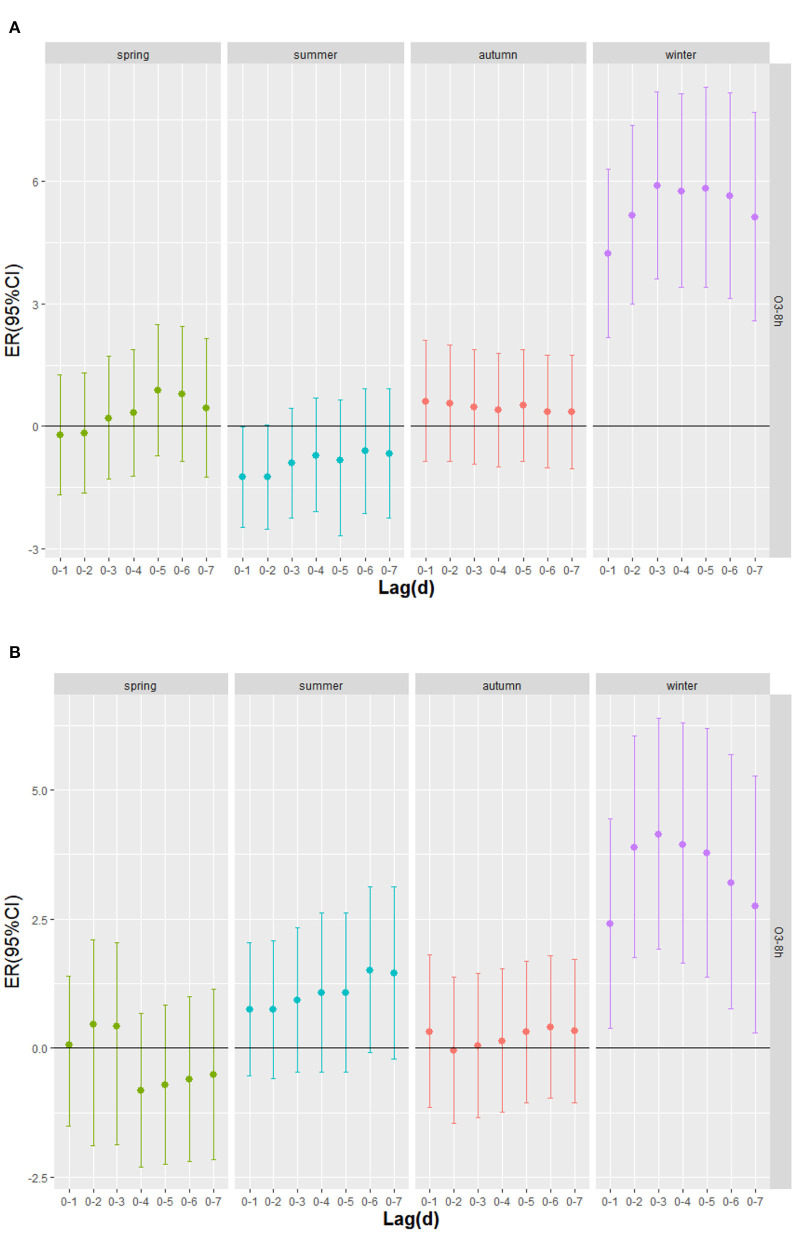
The cumulative excess risk (95%CI) of mortality in season lag-response relationship associated with 10 μg/m^3^ increase of O_3_-8h concentration; **(A)** O_3_-8h lead to respiratory mortality; **(B)** O_3_-8h lead to cardiovascular mortality.

Table 4 shows the excessive risk and 95% confidence intervals of mortality for respiratory diseases and cardiovascular diseases in the optimal lag days in the stratified analysis by age, sex, and season. Results of single-pollutant and multi-pollutant models from respiratory diseases for different age and sex are presented in Figure 5. In the cumulative lag model, for every 10 μg/m^3^ increase of O_3_-8h, the greatest excessive risk of respiratory mortality among people aged <65 years, aged ≥65 years, females and males at lag05, lag05, lag05, lag04 increased by 1.04% (95%CI: −0.45 ~ 2.56%), 1.93%(95%CI: 1.09 ~ 2.78%), 2.17% (95%CI: 0.99 ~ 3.37%), 1.61% (95%CI: 0.76 ~ 2.47%), respectively. When we analyzed the effect of a 10 μg/m^3^ increase in O_3_-8h on cardiovascular diseases, as presented in Figure 6, we saw a similar pattern to respiratory diseases. In this case, the greatest excessive risk of cardiovascular mortality in the cumulative lag model among people aged <65 years, aged ≥65 years, males, and females at lag06, lag03, lag01, lag03 increased by 2.11% (95%CI: 0.31 ~ 3.96%), 1.67%(95%CI: 0.86 ~ 2.48%), 1.44% (95%CI: 0.57 ~ 2.31%), 2.03 (95%C I:1.06 ~ 3.01%), respectively. Except that not statistically significant for age <65 years from respiratory mortality (*P* > 0.05), statistically significant associations were observed in different age and sex groups (*P* < 0.05). In summary, the female group had the highest association with ozone exposure compared to the male group, and the death effect value of respiratory diseases is higher for people age ≥65 years than age <65 years. Conversely, for cardiovascular diseases, the death effect value of people age <65 years is higher than age ≥65 years. Season-specific associations for respiratory and cardiovascular disease in single day lag and cumulative day lag models of O_3_-8h are presented in Figures 7, 8. The strongest effects of O_3_-8h exposure of respiratory mortality in spring, summer, autumn, and winter at lag05, lag06, lag01, and lag03 increased by 0.88% (95%CI: −0.71 ~ 2.49%), −0.61% (95%CI: −2.12 ~ 0.92%), 0.61% (95%CI: −0.86 ~ 2.11%), 5.87% (95%CI: 3.61 ~ 8.18%), respectively. And the effects of O_3_-8h concentration of cardiovascular mortality in spring, summer, autumn, and winter reached the maximum at lag02, lag06, lag06, lag03 increased by 0.45%(95%CI: −1.88 ~ 2.10%), 1.51% (95%CI: −0.09 ~ 3.12%), 0.41% (95%CI: −0.96 ~ 1.79%), 4.14% (95%CI: 1.92 ~ 6.40%), respectively. We did see statistically significant contributions from O_3_-8h on respiratory mortality and cardiovascular mortality in winter (*P* < 0.05). Additionally, the season fluctuation of air pollution demonstrated that O_3_-8h concentration had a stronger association with respiratory mortality in winter and spring and with cardiovascular mortality in summer and winter.

**Table 4:** The ER (95%CI) of respiratory mortality and cardiovascular mortality in the optimal lag days for different age, sex and season.

**Variables**	**Respiratory mortality**		**Cardiovascular mortality**	
	**Lag(d)**	**O3-8h (μg/m3)**	**Lag(d)**	**O3-8h (μg/m3)**
**All**	lag0	1.04 (0.40,1.68)^*^	lag1	1.26 (0.68,1.83)^*^
	lag05	1.77 (0.99,2.57)^*^	lag03	1.68 (0.93,2.45)^*^
**Sex**				
Male	lag0	0.95 (0.24,1.67)^*^	lag1	1.16 (0.48,1.84)^*^
	lag04	1.61 (0.76,2.47)^*^	lag01	1.44 (0.57,2.31)^*^
Female	lag0	1.20 (0.24,2.16)^*^	lag1	1.46 (0.72,2.20)^*^
	lag05	2.17 (0.99,3.37)^*^	lag03	2.03 (1.06,3.01)^*^
**Age(years)**				
≥65	lag1	1.11 (0.51,1.71)^*^	lag1	1.29 (0.68,1.90)^*^
	lag05	1.93 (1.09,2.78)^*^	lag03	1.67 (0.86,2.48)^*^
<65	lag0	1.12 (−0.10,2.35)	lag1	1.39 (0.12,2.67)^*^
	lag05	1.04 (−0.45,2.56)	lag06	2.11 (0.31,3.96)^*^
**Season**				
Spring	lag5	0.91 (0.09,1.73)^*^	lag1	0.24 (−0.75,1.24)
	lag05	0.88 (−0.71,2.49)	lag02	0.45 (−1.88,2.10)
Summer	lag6	0.41 (−0.44,1.27)	lag6	1.12 (0.23,2.02)^*^
	lag06	−0.61 (−2.12,0.92)	lag06	1.51 (−0.09,3.12)
Autumn	lag1	0.52 (−0.54,1.60)	lag5	0.51 (−0.31,1.33)
	lag01	0.61 (−0.86,2.11)	lag06	0.41 (−0.96,1.79)
Winter	lag1	3.71 (2.06,5.39)^*^	lag2	3.07 (1.58,4.58)^*^
	lag03	5.87 (3.61,8.18)^*^	lag03	4.14 (1.92,6.40)^*^

* *P < 0.05*.

## Discussion

In this study, we found that air pollution has an important correlation with residents' health. This paper describes a approach to evaluate the health effect of ozone exposure on respiratory mortality and cardiovascular mortality in Nanchang City from 2014 to 2020. The exposure-response relationship reflects that ozone has an approximately linear positive correlation with the respiratory mortality and cardiovascular mortality. Studies in many cities in the United States, which showed that daily changes in ambient ozone exposure are linked to premature mortality, even at very low pollution level. And they also found robust evidence of the exposure-response relationship between ozone exposure and mortality showed an approximate linear curve (43). Moreover, the single- and two-pollutant models in our studies were constructed to further analyze the lag-response effect of ozone on the risk of death of residents, it was found a significant association for both O_3_-8h and respiratory mortality and cardiovascular mortality with higher effects at the cumulative exposure level.

The results of survey showed that a correlation between O_3_-8h and respiratory mortality and cardiovascular mortality, with percent changes in excessive risk of 1.04% (95%CI: 0.40 ~ 1.68%) and 1.26% (95%CI: 0.68 ~ 1.83%) for a 10 μg/m^3^ increase in O_3_-8h at lag0 and at lag1, respectively. The multi-day moving average lag effect showed that the greatest excessive risk of respiratory mortality and cardiovascular mortality at lag05 and lag03 increased by 1.77% (95%CI: 0.99 ~ 2.57%), 1.68% (95%CI: 0.93 ~ 2.45%), respectively, which is similar to the effect value of related research at home and abroad (42, 44, 45). In our studies, we found that ozone exposure had immediate effect on respiratory mortality and cardiovascular mortality, and the risk of death of cardiovascular diseases was especially susceptible to ozone pollution. But with the cumulative lag effect, it is worth noting that ozone has a greater impact on respiratory mortality. The association between O_3_-8h exposure and respiratory mortality and cardiovascular mortality has been well documented in the epidemiological literature. Chao (41) also found that for every 10 μg/m^3^ increase in O_3_-8h concentration, a single-day lag of 1st-2nd day and multi-day cumulative lag of 3rd day had the greatest effect on cardiovascular disease mortality in Jiangsu Province. Qi's (21) research in Nanjing city discovered that the risk of cardiovascular mortality increased by 1.25% (95%CI: 0.78 ~ 1.72%) for a 10 μg/m^3^ increase in O_3_-8h, while the effect of O_3_-8h on the risk of respiratory mortality was not statistically significant. A study in Sichuan Province (46) consider that for every 10 μg/m^3^ increase in ozone, respiratory mortality would increase by 0.78%(95%CI: 0.12 ~ 1.44%). The same environmental and health cohort study followed up in Canada for 16 years showed that (47), for every 10 μg/m^3^ increase in ozone on respiratory mortality and cardiovascular mortality will increase by 0.97%(95%CI: 0.95 ~ 0.99%) and 1.03% (95%CI: 1.03 ~ 1.05%), respectively. The inconsistency of research results in different regions may be related to temperature (5), population composition, geographical structure and urbanization scale (16, 39, 44). In our studies, in the dual-pollution model, after adjusting for SO_2_, NO_2_, CO, PM_2.5_, and PM_10_, the death risk of respiratory diseases had no significant change, while the death risk of cardiovascular diseases caused by O_3_-8h decreased by 0.23, 0.23, 0.35, 0.38, and 0.27%, respectively. Qi (21) and Yebin (25) discovered that O_3_ had a certain degree of reduction in the risk of cardiovascular mortality after adjusting for PM_2.5_,PM_10_, NO_2_, SO_2_ and CO; Lihong (35) found that after adding PM_2.5_ and NO_2_, the risk of respiratory diseases was not significantly different from the effect value of O_3_ single pollutant, which is consistent with our findings. Furthermore, there was a research showed (48) that after incorporating other pollutants into the single pollutant model, there was no difference in the impact on the health of residents before and after. The reason for the difference may be related to the constructed model, or the single air pollutant may be only considered in the single pollution model effect, and the effect of air pollutant may be affected by the co-linearity or spatial nature of other air pollutants (47).

Regarding gender differences, the effect of ozone exposure on excessive risk of respiratory mortality and cardiovascular mortality in female was greater than in male. It has been proved that females were more likely to suffer from the mortality of respiratory diseases and cardiovascular diseases than males, and females are more susceptible to O_3_-8h than males, which is similar to the results of previous studies at home and abroad (48, 49). The reason for this result may be females have higher gas sensitivity, shorter respiratory tract and more susceptible to air pollutants compared with males (5, 37, 44). However, there is also evidence that males are more vulnerable to the effects of ozone on respiratory mortality than females. A study conducted in Li et al. (50) explained that pneumonia and bronchitis are more common in males with a history of smoking and exposure to different occupations, which may exacerbate the impact of O_3_-8h on respiratory mortality in males. Mechanistically, as a result of reduced uptake of ozone into conducting airways, cigarette smoking may shift the longitudinal distribution of ozone uptake distally toward the respiratory airways, thereby leading to respiratory diseases in the population (51). However, there is no detailed study on whether the smoking rate of adults will affect the association between ozone exposure and cardiovascular diseases. Our studies have also exhibited that the elevated concentration of O_3_-8h significantly increased the risk of respiratory mortality among residents aged ≥65 years (compared to aged <65 years), which is consistent with the results of previous studies (5, 19–22, 44). The structure of the respiratory system changes as we age, with decreased chest wall compliance, respiratory muscle strength, and vital capacity, which could lead to the risk of death of respiratory diseases in older people (52). As for the risk of cardiovascular mortality, the effect value of residents aged <65 years is higher than that of residents aged ≥65 years, which is inconsistent with previous researches found that people aged ≥65 years were more closely related to cardiovascular mortality risks (37, 48, 50). On the one hand, the younger with these cardiometabolic conditions had a higher prevalence of cardiovascular diseases in association with higher air pollution exposures than individuals without these cardiometabolic conditions (53). On the another hand, some scholars detected stronger associations between air pollutants and cardiometabolic risk factors in younger participants and for those with a family history of cardiovascular diseases (54). In summary, ozone exposure is closely related to the risk of respiratory mortality and cardiovascular mortality in people of different ages and sexes. Among them, females and people aged ≥65 years are sensitive groups. Nevertheless, ozone as an important air pollutant, we also should also be paid attention to the health risk of people aged <65 years because they are more closely linked to cardiovascular mortality for a 10 μg/m^3^ increase in O_3_-8h.

Climate change has a negative impact on human health due to increased exposure to adverse climate-related stresses (55). According to the climatic characteristics and the analysis of daily average temperature and daily average relative humidity of Nanchang City during the study period, it is known that the higher the temperature in the warm season (spring, summer), the greater the humidity. When considering differential effects by season, it had been reported that seasonal changes will affect the impact of air pollutants on human health (56). We conducted that season-specific associations for the mortality risk of respiratory diseases showed stronger associations in winter and spring for a 10 μg/m^3^ increase in O_3_-8h, which is in agreement with the finding of the study by Yuqi (42) in Lishui district. And the risk of cardiovascular mortality was stronger associated with O_3_-8h in summer and winter. In summer, ozone precursors in the air produce ozone more quickly with the increase of temperature (57). The reduced levels of nitrogen dioxide and carbon monoxide in summer months can be attributed to the contribution of these compounds to photochemical reactions occurring under the influence of solar radiation which result in the formation of ozone (58). Most importantly, we indicated the excessive risk of ozone pollution on respiratory mortality and cardiovascular mortality occurred earlier and had a greatest effect in winter than in the warm season. Higher levels of ozone pollution in winter months may also be associated with increased low emissions from local home furnaces, as well as more frequent in these periods, inversion of temperature resulting in smog events (59).

This study has several limitations that should be addressed. First, the air pollution exposure data of the study is the monitoring point data, which may be affected by distance, climate and other reasons, and cannot fully reflect the individual exposure (39, 40, 48), may overestimate the impact of air pollutants on the death of residents. Second, there exists another source of misclassification of ozone concentrations as this study did not use personal ozone exposure, because some personal behavior factors were not taken into consideration, such as air conditioner use, and time spent outdoors (60). Third, this study was conducted in a single city (Nanchang), thus, our findings cannot be generalized to other cities with different environmental and economic characteristics. Finally, the research only examined the correlation of respiratory mortality and cardiovascular mortality and ozone pollution, we were not able to involve more diseases, which is also a limitation of this study. As an important pollutant, ozone has independent health hazards. According to the source and changes of ozone in Nanchang city, government departments should strengthen the monitoring and control of ozone pollutant emissions, actively formulate energy-saving and emission reduction measures for ozone to reduce the heavy burden. In this vein, government departments also should promote actions and measures to enhance numerous aspects around the subject. Boosting education, training and public participation are some of the relevant actions for maximizing the opportunities to achieve the targets and goals on the crucial matter of ozone pollution. Without any doubt, technological improvements makes our world easier and it seems difficult to reduce the harmful impact caused by gas emissions, we could limit its use by seeking reliable approaches (61). When carrying out the prevention and control of ozone pollution, the relationship between PM_2.5_ and ozone is often discussed. In winter, PM_2.5_ treatment should be carried out, and in summer, the synergy between ozone and PM_2.5_ should be explored. As a secondary pollutant, ozone has a complex reaction mechanism, and it is necessary to implement multi-target and multi-pollutant coordinated control. In the entire prevention and control work, more emphasis should be placed on the importance of atmospheric oxidation (62). Last but not least, taking appropriate protective measures for the entire population, sensitive groups, and high-risk groups to improve residents' awareness of self-protection. At the same time, identifying vulnerable subpopulations and the impact of ozone on these subpopulations will help in establishing air quality standards that will better protect these groups (53). A study (63) have shown that masks containing activated carbon interlayer have good protection against ozone at different pollution levels.

## Conclusion

This study provides evidence of evaluating the effects of O_3_-8h exposure on respiratory mortality and cardiovascular mortality in Nanchang city from 2014 to 2020. To our knowledge, results confirm that ozone pollution would increase the risk of respiratory mortality and cardiovascular mortality. Our findings complement previous studies that are lacking by revealing that ozone pollutants have a lag effect on the health of the population in Nanchang city, China. With the rapid of economic growth and the development of processing industries such as electric power, gas and non-ferrous metal smelting in Nanchang city, the government should introduce corresponding control policies, take actions to reduce air pollution and make interventions for sensitive individuals to improve our health.

## Data Availability Statement

The original contributions presented in the study are included in the article/supplementary material, further inquiries can be directed to the corresponding author.

## Author Contributions

HW and JF conceived and designed the work. HW led the study, carried out the time-series studies, analyzed the data, and approved the version to be published. KL involved in the study design and the interpretation of the results. JF helped to conceptualize the study, provided intellectual advice, and revise various drafts of the manuscript. All authors read and approved the final manuscript.

## Funding

This work was funded by Science and Technology Plan of Jiangxi Health Commission (20204847).

## Conflict of Interest

The authors declare that the research was conducted in the absence of any commercial or financial relationships that could be construed as a potential conflict of interest.

## Publisher's Note

All claims expressed in this article are solely those of the authors and do not necessarily represent those of their affiliated organizations, or those of the publisher, the editors and the reviewers. Any product that may be evaluated in this article, or claim that may be made by its manufacturer, is not guaranteed or endorsed by the publisher.

## References

[1] Fengping H Yongming G. Health impacts of air pollution in China. Front Environ Sci Eng. (2021) 15:74–92. 10.1007/s11783-020-1367-1

[2] World Health Organization. Ambient (Outdoor) Air Pollution (2018). https://www.who.int/news-room/fact-sheets/detail/ambient-(outdoor)-air-quality-and-health (accessed 22, 2018).

[3] World Health Organization. Ambient air pollution: A global assessment of exposure and burden of disease (2016). https://apps.who.int/iris/handle/10665/250141 (accessed 13, 2016).

[4] Qiang Z Yixuan Z Dan T Min S Shuxiao W Yuanhang Z. Drivers of improved PM^2.5^ air quality in China from 2013 to 2017. Proc Natl Acad Sci. (2019) 116:24463–9. 10.1073/pnas.1907956116 31740599 PMC6900509

[5] Mireadili K Yizaitiguli W Fan F Ye L Wei Q Anthony JD. Spatio-temporal patterns of air pollution in China from 2015 to 2018 and implications for health risks. Environ Pollut. (2020) 258:113659. 10.1016/j.envpol.2019.113659 31806463

[6] Yihui L. The status quo of ozone pollution in China and recommendations for management and control measures. Shandong Chem Ind. (2021) 50:253–4. 10.19319/j.cnki.issn.1008-021x.2021.01.112

[7] Huan L Tiantian H Meng W. Impact of air pollution on residents' medical expenses: a study based on the survey data of 122 cities in China. Front Public Health. (2021) 9:743087. 10.3389/fpubh.2021.743087 34988046 PMC8720779

[8] Department of ecological environment. China Ecological Environment Status Bulletin 2020 (Excerpt). Environ Protec. (2021) 49:47–68. 10.14026/j.cnki.0253-9705.2021.11.010

[9] Xiao L Jiayun H Lin Z Owen RC Martin GS Xiaobin X. Severe surface ozone pollution in China: a global perspective. Environ Sci Technol Lett. (2018) 5:487–94. 10.1021/acs.estlett.8b0036635298883

[10] Sahu SK Shuchang L Song L Dian D Jia X. Ozone pollution in China: Background and transboundary contributions to ozone concentration & related health effects across the country. Sci Total Environ. (2021) 761:144131. 10.1016/j.scitotenv.2020.144131 33352350

[11] Tao W Likun X Peter B Yunfat L Li L Li Z. Ozone pollution in China: A review of concentrations, meteorological influences, chemical precursors, and effects. Sci Total Environ. (2016) 575:1582–96. 10.1016/j.scitotenv.2016.10.081 27789078

[12] Kan C Ming Y Junshan W Bingwei C Xianfeng C Zilu H. Characteristics of ozone pollution in Jiangxi Province and its relationship with meteorological factors. Environ Monit Chin. (2021) 37:44–59. 10.19316/j.issn.1002-6002.2020.02.06

[13] Chunlian L Chunyan H. Characteristics of ambient air of ozone pollution in Nanchang city and its influencing factors. Jiangxi Chem Ind. (2016) 06:109–10. 10.14127/j.cnki.jiangxihuagong.2016.06.035

[14] Yingying H Chen C Hongyan B Jin S. Analysis on the source and sensitivity of ozone in the southwestern part of the Pearl River Delta in spring. J Ecolog Environ. (2021) 30:984–94. 10.16258/j.cnki.1674-5906.2021.05.011

[15] Chao C Xu L Hui Y Renchang Y Xiaoting Q. Analysis on the source and sensitivity of ozone in the southwestern part of the Pearl River Delta in spring. Environ Moni China. (2019) 35:73–81. 10.19316/j.issn.1002-6002.2019.03.11

[16] Qing Y Zhiqiang M Tianyi H Wenyan F Xu Y Yingxiao T. Spatial and temporal distribution characteristics of ozone and estimation of background concentration the Beijing-Tianjin-Hebei region. China Environ Sci. (2021) 41:4999–5008. 10.19674/j.cnki.issn1000-6923.20210706.007

[17] Xianyu Y Jiajun Y Yaqiong L Zhihong L Shigong W Shihua L. Characteristics and formation mechanism of a severe O^3^ episode in Chengdu and surrounding areas. China Environ Sci. (2020) 40:2000–9. 10.19674/j.cnki.issn1000-6923.2020.0226

[18] Chang Howard H Jingwen Z Montserrat F. Impact of climate change on ambient ozone level and mortality in southeastern United States. Int J Environ Res Public Health. (2010) 7:2866–80. 10.3390/ijerph7072866 20717546 PMC2922733

[19] Weiheng H Bingyu C Ho K Yasushi H Yue LG. Significant effects of exposure to relatively low level ozone on daily mortality in 17 cities from three Eastern Asian Countries. Environ Res. (2019) 168:80–4. 10.1016/j.envres.2018.09.017 30278365

[20] Weipeng Y Miaomiao L Jun B. Meta-analysis study on the relationship between short-term ozone exposure and population mortality in China. Acta Sci Circumstant. (2020) 40:2644–51. 10.13671/j.hjkxxb.2020.0146

[21] Qi C Hong S Xiaodong C Zheng D. Acute health impacts of ozone exposure on daily mortality in Nanjing. Jiangsu J Prev Med. (2017) 28:366–8. 10.13668/j.issn.1006-9070.2017.04.02

[22] Quan Z Tingting S Shuling K Xin L. Health effects of ozone exposure on non-accidental deaths in Fuzhou. Chin J Prevent Med. (2021) 22:218–22. 10.16506/j.1009-6639.2021.03.012

[23] Zhaozhong F Alessandra DM Alessandro A Maurizio G Pierre S Hanqin T. Economic losses due to ozone impacts on human health, forest productivity and crop yield across China. Environ Int. (2019) 131:104–13. 10.1016/j.envint.2019.104966 31284106

[24] Yu S Zhiwei S Junji C Xinming W Liuju Z Xinhui B. Systematic review of Chinese studies of short-term exposure to air pollution and daily mortality. Environ Int. (2013) 54:100–11. 10.1016/j.envint.2013.01.010 23434817

[25] Yebin T Wei H Xiaoliang H Liuju Z Shou EL Yi L. Estimated acute effects of ambient ozone and nitrogen dioxide on mortality in the Pearl River Delta of southern China. Environ Health Perspect. (2012) 120:393–8. 10.1289/ehp.1103715 22157208 PMC3295344

[26] Chit-Ming W Nuntavarn VV Haidong K Zhengmin Q. Public health and air pollution in Asia (PAPA): a multicity study of short-term effects of air pollution on mortality. Environ Health Perspect. (2008) 116:1195–202. 10.1289/ehp.11257 18795163 PMC2535622

[27] Kamal JM Anil N. Continuous increases of surface ozone and associated premature mortality growth in China during 2015-2019. Environ Pollut. (2021) 269:116183. 10.1016/j.envpol.2020.116183 33288298

[28] Marine LC Luc Z. Ozone atmospheric pollution and Alzheimer's disease: from epidemiological facts to molecular mechanisms. J Alzheimer's Dis. (2018) 62:503–21. 10.3233/JAD-170857 29480184

[29] Yi L Yu S Canjun Z Zhiqiang M. Estimated acute effects of ozone on mortality in a rural district of Beijing, China, 2005-2013: a time-stratified case-crossover study. Int J Environ Res Public Health. (2018) 15:2460–71. 10.3390/ijerph15112460 30400565 PMC6266742

[30] Drew BD Jianbang X Jinhan M Feng L Mingkei C Jicheng G. Association of ozone exposure with cardiorespiratory pathophysiologic mechanisms in healthy adults. JAMA Internal Med. (2017) 177:1344–53. 10.1001/jamainternmed.2017.2842 28715576 PMC5710579

[31] Sanjay R Sadeer GAI-K Robert DB. Air pollution and cardiovascular disease: JACC state-of-the-art review. J Am Coll Cardiol. (2018) 72:2054–70. 10.1016/j.jacc.2018.07.099 30336830

[32] Mengjia X Dong L Jinfeng G. Analysis and suggestions on ecological environmental protection planning indicators for the “14th Five-Year Plan”. Environ Ecol. (2019) 1:27–32. Available online at: https://kns.cnki.net/kcms/detail/detail.aspx?FileName=HJSX201906009&DbName=CJFQ2019

[33] Jun C. China's climate change targets are clearer. China Today. (2021) 70:57–9. Available online at: https://kns.cnki.net/kcms/detail/detail.aspx?FileName=JRZZ202101022&DbName=CJFN2021

[34] Jiangxi will focus on ozone pollution control during the 14 th Five-Year Plan period. Jiangxi Building Mater. (2021) 03:285. Available online at: https://kns.cnki.net/kcms/detail/detail.aspx?FileName=JXJC202103197&DbName=CJFQ2021

[35] Lihong Z Zedong Y Peng P Xunzhi OY. An analysis of the terrain gradient effect on landscape informational atlas in Nanchang city, China. Acta Agric Univ Jiangxie nsis. (2016) 38:767–75. 10.13836/j.jjau.2016110

[36] JinWu Z HE. Hong, Academician of the Chinese Academy of Engineering: Collaborative control of particulate matter and ozone pollution has become a challenge. Chinese J Sci. (2021) 4:1. 10.28514/n.cnki.nkxsb.2021.003173

[37] Yunfei J Chunjing C Yanzhao T Fengxia S Li W. Association between ambient ozone and circulatory mortality in Nanjing:a time-series analysis. J Environ Health. (2020) 37:42–6. 10.16241/j.cnki.1001-5914.2020.01.010

[38] Francesca D. Time-series analysis of air pollution and mortality: a statistical review. Res Rep Health Eff Inst. (2004) 123:29–33. 15757000

[39] Chris CL Richard BH Jiyoung A Yongzhao S Debra TS Rena RJ. Long-term exposure to ozone and cause-specific mortality risk in the United States. Am J Respirand Crit Care Med. (2019) 200:1022–31. 10.1164/rccm.201806-1161OC 31051079 PMC6794108

[40] Michelle LB Antonella Z Francesca D. Evidence on vulnerability and susceptibility to health risks associated with short-term exposure toparticulate matter: a systematic review and meta-analysis. Am J Epidemiol. (2013) 178:865–76. 10.1093/aje/kwt090 23887042 PMC3775545

[41] Chao Z Rui D Changchun X Yachun X Han C Furong Z. Association between air pollution and cardiovascular mortality in Hefei, China: a time-series analysis. Environ Pollut. (2017) 229:790–7. 10.1016/j.envpol.2017.06.022 28797522

[42] Yuqi C Zhigang J Ping C Lijun F Xudan Z Yuepu P. Short-term effect of fine particulate matter and ozone on non-accidiental-mortality and respiratory mortality in Lishui district, China. BMC Public Health. (2021) 21:1661–72. 10.1186/s12889-021-11713-9 34517854 PMC8439017

[43] Michelle LB Roger DP Francesca D. The exposure-response curve for ozone and risk of mortality and the adequacy of current ozone regulations. Environ Health Perspec. (2006) 114:532–6. 10.1289/ehp.8816 16581541 PMC1440776

[44] Murray CJL Aravkin AY Zheng P Abbafati C Abbas KM Abbasi-Kangevari M. Global burden of 87 risk factors in 204 countries and territories, 1990-2019: a systematic analysis for the global burden of disease study 2019. Lancet. (2020) 396:1223–49. 10.1016/S0140-6736(20)30752-2 33069327 PMC7566194

[45] Tiantian L Meilin Y Wenjun M Jie B Tao L Huangliang L. Short-term effects of multiple ozone metrics on daily mortality in a megacity of China. Environ Sci Pollut Res Int. (2015) 22:8738–46. 10.1007/s11356-014-4055-5 25572272

[46] Bing G Fei C Ying D Hongliang Z Xue Q Zhijiao Q. Using rush hour and daytime exposure indicators to estimate the short-term mortality effects of air pollution: a case study in the Sichuan Basin, China. Environ Pollut. (2018) 242:1291–8. 10.1016/j.envpol.2018.08.028 30121483

[47] Dan LC Paul AP Perry H Jeffrey RB Aaronvan D Randall VM. Ambient PM^2.5^, O^3^, and NO^2^ exposures and associations with mortality over 16 years of follow-up in the Canadian census health and environment cohort (CanCHEC). Environ Health Persp. (2015) 123:1180–6. 10.1289/ehp.1409276 26528712 PMC4629747

[48] Turner MC Jerrett M Pope CA III Krewski D Gapstur SM Diver WR. Long-term ozone exposure and mortality in a large prospective study. Am J Respir Crit Care Med. (2016) 193:1134–42. 10.1164/rccm.201508-1633OC 26680605 PMC4872664

[49] Jixiang X. Study on the association between ambient air pollution and daily non-accidental deaths in Hefei, during 2013-2017: a time-series study. [master's thesis]. [Anhui(IL)]: Anhui Medicine University. (2020)

[50] Li W Yu B Fengying Z Wuyi W Xiaojian L Thomas K. Spatiotemporal patterns of ozone and cardiovascular and respiratory disease mortalities due to ozone in Shenzhen. Sustainability-B asel. (2017) 9:559. 10.3390/su9040559

[51] Melissa LB Timothy MB Abdellaziz BJ Rebecca B. James SU. Longitudinal distribution of ozone absorption in the lung: Comparison of cigarette smokers and nonsmokers. Toxicol Appl Pharmacol. (2009) 236:270–5. 10.1016/j.taap.2009.02.006 19233220

[52] Jean PJ. Aging of the respiratory system: impact on pulmonary function tests and adaptation to exertion. Clin Chest Med. (2005) 26:469–84. 10.1016/j.ccm.2005.05.004 16140139

[53] Michelle LB Antonella Z Francesca D. Who is more affected by ozone pollution? A systematic review and meta-analysis. Am J Epidemiol. (2014) 180:15–28. 10.1093/aje/kwu115 24872350 PMC4070938

[54] Boyi Y Yuming G Lana M Zhengmin Q Michael SB Joachim H. Association of long-term exposure to ambient air pollutants with risk factors for cardiovascular disease in China. JAMA Network Open. (2019) 2:e190318. 10.1001/jamanetworkopen.2019.0318 30848806 PMC6484675

[55] Rajendra KP Myles RA Vicente RB John B Wolfgang C Renate C. Climate change 2014: synthesis report. Contribution of Working Groups I, II and III to the fifth assessment report of the Intergovernmental Panel on Climate Change: IPCC. (2014). p. 151.

[56] Chaicharn P Warawut C Chalerm L Chaiwat B Athavudh D Theerakorn T. Acute effects of air pollutants on daily mortality and hospitalizations due to cardiovascular and respiratory diseases. J Thorac Dis. (2019) 11:3070–83. 10.21037/jtd.2019.07.37 31463136 PMC6687987

[57] Lijie Q Jianqin G Shijie L Fang F Weimin B Xu L. Seasonal association between ambient ozone and mortality in Zhengzhou, China. Int J Biometeorol. (2017) 61:1003–10. 10.1007/s00484-016-1279-8 27981338

[58] Jez M. Ochrona atmosfery. Warszawa: Oficyna Wydawnicza Wyzszej Szkoły Ekologii i Zarzadzania. (2009).

[59] Lei Z Hengrui L Fengying C Zi C Weijie G Jianhua L. Association between air pollution and cardiovascular mortality in China: a systematic review and meta-analysis. Oncotarget. (2017) 8:66438–48. 10.18632/oncotarget.20090 29029525 PMC5630425

[60] Hwashin HS Rajendra PP Aubrey M Marc SD. Temporal trends in associations between ozone and circulatory mortality in age and sex in Canada during 1984-2012. Sci Total Environ. (2020) 724:137944. 10.1016/j.scitotenv.2020.137944 32408420

[61] Loannis M Elisavet S Agathangelos S Eugenia B. Environmental and health impacts of air pollution: a review. Front Public Health. (2020) 8:14. 10.3389/fpubh.2020.00014 32154200 PMC7044178

[62] Xiaofang L. Academician Yuanhang Zhang: Atmospheric oxidation and prevention and control of ozone pollution. High-Technol Commercializ. (2022) 28:20–3. Available online at: https://kns.cnki.net/kcms/detail/detail.aspx?FileName=GKFC202201017&DbName=DKFXTEMP

[63] Yunpu L Qian L Jingjing C Na L Chunyu X Dongqun X. Filtration effects of commonly uesd masks on ozone. J Environ Health. (2018) 35:947–50. 10.16241/j.cnki.1001-5914.2018.11.002

